# Hepatitis C treatment access and uptake for people who inject drugs: a review mapping the role of social factors

**DOI:** 10.1186/1477-7517-10-7

**Published:** 2013-05-07

**Authors:** Magdalena Harris, Tim Rhodes

**Affiliations:** 1 Centre for Research on Drugs and Health Behaviour, London School of Hygiene and Tropical Medicine, 15-17 Tavistock Place, London, WC1H9SH, UK

**Keywords:** Hepatitis C, Antiviral treatment, Treatment access, People who inject drugs, Social factors

## Abstract

**Background:**

Evidence documents successful hepatitis C virus (HCV) treatment outcomes for people who inject drugs (PWID) and interest in HCV treatment among this population. Maximising HCV treatment for PWID can be an effective HCV preventative measure. Yet HCV treatment among PWID remains suboptimal. This review seeks to map social factors mediating HCV treatment access.

**Method:**

We undertook a review of the social science and public health literature pertaining to HCV treatment for PWID, with a focus on barriers to treatment access, uptake and completion. Medline and Scopus databases were searched, supplemented by manual and grey literature searches. A two step search was taken, with the first step pertaining to literature on HCV treatment for PWID and the second focusing on social structural factors. In total, 596 references were screened, with 165 articles and reports selected to inform the review.

**Results:**

Clinical and individual level barriers to HCV treatment among PWID are well evidenced. These include patient and provider concerns regarding co-morbidities, adherence, and side effect management. Social factors affecting treatment access are less well evidenced. In attempting to map these, key barriers fall into the following domains: social stigma, housing, criminalisation, health care systems, and gender. Key facilitating factors to treatment access include: combination intervention approaches encompassing social as well as biomedical interventions, low threshold access to opiate substitution therapy, and integrated delivery of multidisciplinary care.

**Conclusion:**

Combination intervention approaches need to encompass social interventions in relation to housing, stigma reduction and systemic changes in policy and health care delivery. Future research needs to better delineate social factors affecting treatment access.

## Review

Worldwide 170 million people are estimated to live with chronic HCV [1], with annual mortality rates due to HCV-related liver diseases estimated at over 350 000 [2]. Global HCV antibody prevalence among people who inject drugs (PWID) is high. A recent systematic review, for instance, identified 24 countries where HCV antibody prevalence in PWID ranged from 40–60% (such as Australia, UK, Greece), 25 countries with a range of 60-80% (USA, Canada, Germany) and 12 (Mexico, Thailand, Russia, the Netherlands) where prevalence was 80% or higher [3]. The World Health Organisation identifies PWID as a key target group for HCV prevention and treatment [2].

HCV antiviral treatment with peginterferon alfa and ribavirin is the standard of care for chronic HCV, with a 50-85% cure rate depending on genotype [4]. Modelling studies indicate that scaling-up HCV treatment access to PWID, specifically current injectors, has the potential to reduce the pool of communicable disease in the population, acting as an effective preventative measure [5,6]. Qualitative [7,8] and quantitative research [9-12] shows that PWID are interested in assessing and commencing HCV treatment. PWID have rates of HCV treatment adherence and successful completion comparable to other populations [13-16]. Clinical guidelines in a number of countries (such as the UK, Australia, US, Canada, France) have been modified to remove current injecting drug use as HCV treatment exclusion criteria [17-20]. Yet HCV treatment uptake among PWID is suboptimal, and estimated to be in the order of 2-4% of those eligible [5,17,21,22]. Why might this be so? What interplay of factors mediate HCV treatment access for PWID? And what specifically, might be the role of social and structural factors?

Drawing upon published literature pertaining to HCV treatment as well as HCV among PWID, we seek to map the social factors potentially mediating HCV treatment access and uptake for PWID. This will assist in developing a descriptive typology of social factors and how they function potentially as ‘barriers’ and ‘facilitators’ to treatment access. This is necessary because there is a dearth of pooled evidence investigating the role of social factors affecting HCV treatment. We therefore draw upon previous work on the conditionality of HIV treatment access for PWID as a way of conceptualising ‘social factors’ and their relevance [23-25]. This work on HIV treatment maps the treatment access environment as a product of interplay between *macro-level factors* (such as the adverse impacts of criminalisation, social and material inequality, and health policy) *and meso-level factors* (such as related to systems of service administration, management and delivery) [26]. Given the absence of previous review on social factors affecting HCV treatment, this review takes a two step approach to two different literatures in an attempt to map the field. First, we draw upon literature specific to HCV treatment for PWID, generated through a Medline and Scopus search of published papers since the year 2000, selected for their relevance to barriers and facilitators to treatment access. Second, we draw upon literature specific to HCV among PWID, generated through a Medline and Scopus search of published papers since the year 2000, and selected for their relevance regarding ‘social factors’ linked to HCV risk, prevention or treatment. The first of these literatures (HCV treatment) focuses predominantly on clinical and individual level factors, and the second (HCV among PWID) focuses on social and system level factors with a heavier focus on qualitative studies.

## Method

Our review comprised two steps. The first step involved a search of the literature pertaining to HCV treatment for PWID. The second step sought to map the role of social factors in HCV treatment access. The searches were conducted on Medline and Scopus databases and limits were set for publications between 2000 and 2011 (inclusive), in English language.

### Step 1

**Figure 1:**
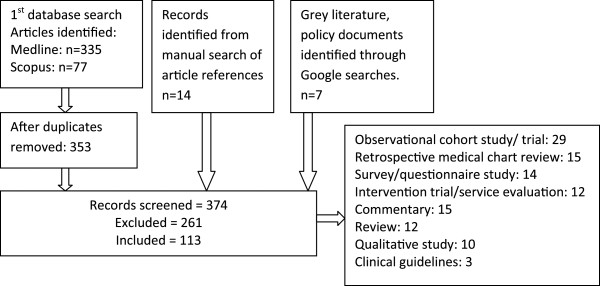
Search strategy one.

The Medline database was searched using a combination of indexed subject headings: (hepatitis C) AND (Interferons OR treatment mp.) AND (methadone OR Opiate Substitution Treatment OR substance abuse, intravenous). Indexed subject headings were broadened using the ‘explode’ operator and the addition of ‘treatment’ as a key word was added. 335 articles were identified. The Scopus database search, using the combination of keywords: (hepatitis C OR HCV) AND (interferon OR treatment) AND (methadone OR opiate substitution therapy OR inject* OR intravenous), yielded 77 articles. Articles totalled 353 after removal of duplicates. A manual search of article bibliographies yielded 14 records, and seven were identified through a Google search of the grey and policy literature. The resulting 374 documents were screened for relevance to HCV treatment barriers and facilitators. Articles were excluded if their primary focus was on HCV transmission, prevalence, incidence, risk factors or prevention. Also excluded were articles which focused on HCV treatment trial design and included no outcome measures. Where multiple articles drew on data from the same research cohort and reported similar findings, the most relevant article was selected. This screening process resulted in 113 articles which were read in full and analysed for data specific to HCV treatment barriers and facilitators (Figure 1).

The majority of the articles resulting from the first search strategy reported on the outcomes of observational cohort studies or clinical trials (n=29), followed by retrospective medical chart reviews (n=15) and quantitative questionnaire studies (n=14). Articles primarily focused on HCV treatment management, adherence and successful completion in PWID (n=29). Other areas addressed included HCV treatment eligibility, access and outcomes for people with co-morbidities (n= 13) or HIV (n=7), rates of re-infection (n=7), integrated care (n=11), and the knowledge and attitudes of PWID towards HCV treatment (12). Social structural barriers to treatment access were rarely mentioned apart from two articles which addressed stigma [27,28], and one which commented on geographic access to treatment [29].

### Step 2

We honed the focus of our review in the second step, focusing specifically on the identification of social structural barriers to HCV treatment access for PWID. Informed by conceptualisations of social and structural factors and risk and enabling environments in relation to HIV [24,25,30], Medline was searched with the following indexed subject headings and keywords (where applicable): (HCV or hepatitis C) AND (substance abuse, intravenous) AND (criminal* OR prison* OR homeless* OR accommodation OR gender OR poverty OR social marginalisation OR social stigma). This, with the 2000–2011 timeframe and an English language limit, identified 182 articles. The Scopus database was searched using the combination of keywords (hepatitis C OR HCV) AND (inject* OR methadone) AND (interferon OR treatment) AND (stigma OR discrimination OR poverty OR social marginalisation OR gender OR homeless* OR housing OR accommodation OR criminal* OR prison*). This, with the publication time limits of 2000 to 2011 inclusive, yielded 54 articles. After duplicates were removed, including duplicates with search number 1, the number of articles totalled 216. An additional six articles were identified from a manual search of article references, resulting in 222 articles screened with 170 excluded.

**Figure 2:**
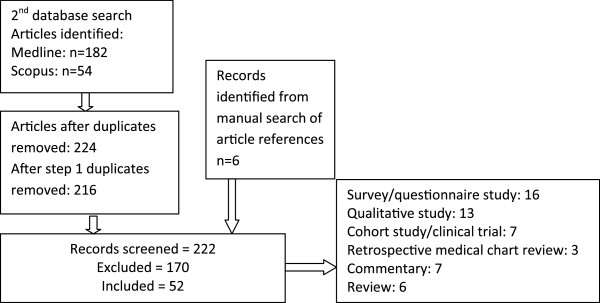
Search strategy two.

Articles were included if they contained data relating to social structural components pertaining to HCV treatment access. The majority of the articles resulting from the second search strategy reported on the outcomes of survey studies (n=16), with nine of these incorporating HCV antibody testing. Twelve studies were qualitative and seven reported on the outcomes of cohort studies. Primary areas of focus included prison populations (n=18), homelessness (n=14), stigma (n=9), gender (n=6) and social exclusion/poverty (n=3). Secondary analysis of all articles from both searches identified health care systems as an additional structural barrier (Figure 2).

## Results

### Individual level factors

The extant literature on HCV treatment primarily focuses on clinical and individual level factors, such as complications posed by co-morbidities, treatment side-effects, efficacy and adherence, and HCV re-infection potentials. Research capturing how HCV treatment is experienced emphasises the potential adverse effects of treatment on physical, psychological and social well-being. It is common, for example, for participants to describe stresses on interpersonal relationships, compromised ability to partake in social, caring and work roles, as well as changes to mood, appearance, demeanour and self-concept after treatment commencement [31-35]. For people considering treatment, side-effects are a primary concern [9,17,31,36,37], as is lengthy treatment duration and uncertain efficacy [36]. For PWID who need to prioritise acute health and social concerns over preventative health care [27,38], who have caring responsibilities [39], and for whom the effects of living with HCV may be relatively unproblematic [8,37,40], there may be little incentive to commence treatment.

Research indicates that HCV treatment fears, or disinterest, at the level of individual PWID can be exacerbated by: low patient and provider treatment literacy [9,27,41-43]; fear of medical investigations, particularly involving biopsy and phlebotomy [37,40,44,45]; concerns about potential relapse to, or exacerbation of, injecting drug use [31]; communication problems with providers [36,46]; and previous stigmatising and negative experiences with health care systems and providers [27,40,45,47,48]. At the outset then, it is clear that individual level concerns have a context, in that they interplay with both social as well as systemic factors (such as stigma and mistrust of treatment delivery systems).

Despite these individual level concerns, there is evidence of interest in treatment uptake among PWID [9,11]. This interest may, however, be thwarted by provider reticence to treat PWID. Provider reticence is documented as stemming from: concerns about treatment adherence among PWID [49,50]; the impact of psychiatric co-morbidities [51], HIV co-infection [37,52] and/or current alcohol and drug consumption [13,53,54]; as well as potential HCV re-infection [55,56]; and a presumption of a lack of interest from clients [39]. A growing body of literature evidences that many of these provider concerns should not preclude consideration for HCV treatment, with: adherence among cohorts of PWID equalling that of other patient groups [14,16,57]; low re-infection occurrences [55,56,58]; treatment successes among current drug and alcohol users [13,14,16,22,53,59-61]; as well as those with psychiatric co-morbidities [51,62,63] and HIV [64-66]. While HCV treatment can be complicated by HIV comorbidity, including antiretroviral drug-drug interactions and co-occurring antiviral toxicity [65,67,68], a 48 week treatment with peginterferon plus ribavirin for all genotypes has been found to be effective in co-infected individuals, including for PWID [65,69].

A primary area of concern identified in the literature is psychiatric co-morbidity among PWID, including major depression, anxiety, posttraumatic stress and bipolar disorders. PWID may engage in substance use as a form of self medication in response [70]. Reticence to treat PWID with psychiatric conditions is understandable, given the neuropsychiatric adverse effects associated with interferon treatment, including impairment in concentration, depression, insomnia, and irritability [71,72]. Yet people with psychiatric histories can adhere to and complete HCV treatment at rates as high as others, if their mental health status is closely monitored and treated [51,62,72]. This may involve prophylactic antidepressant therapy before beginning HCV treatment in patients thought to have a high risk of depression [51,72]. Taken together, evidence suggests the need for caution when commencing PWID with psychiatric co-morbidities on HCV treatment.

## Social level factors

Individual level factors affecting treatment decision making are shaped by, as well as reproduce, the social contexts in which efforts to access treatment are made. Treatment access, as with patterns of health behaviour more broadly, can therefore be seen as a product of individual and environment interactions [30]. The literature accessed in the second step of our search points to a number of key domains potentially relevant for mapping how social factors affect HCV treatment access. These include: stigma and discrimination; housing; geography; criminalisation; health care systems; gender; and culture. We note the parallels here with evidence concerning the conditionality of HIV treatment access among PWID [24-26].

### Stigma and discrimination

Stigma and discrimination are well evidenced barriers to HCV testing and treatment access among PWID [27,40,44,45,48,61,73]. The health care setting is reported as the most common site of experienced HCV discrimination by PWID, potentially due to the enhanced likelihood or necessity of HCV status disclosure in this environment [27,40,73,74]. Experiences of discriminatory treatment by health care providers can be a barrier to future health care access for PWID [38], also impacting on HCV testing and treatment uptake [27,40,45,61]. Fears of confidentiality breaches by health care providers in relation to HCV status, and the resulting discrimination this may provoke, have been identified as an additional barrier to HCV testing and treatment uptake and adherence [44,75] including in the prison setting [76]. Stigma is reported to be experienced particularly acutely by female PWID [40,73,77] and can be a barrier to healthcare seeking by PWID in rural communities who face a limited choice of health care providers [78].

HCV disclosure decisions are impacted by experiences or expectations of stigma and discrimination [28,44,47]. Health status disclosure creates or constrains avenues for support, especially in the context of HCV treatment [79]. While there has been little HCV-specific research addressing this issue, studies of HIV treatment have shown that individuals who do not disclose their HIV status to others in their social networks often display reduced levels of HIV medication adherence [80,81]. At a macro level, stigma related to injecting drug use and HCV can result in political inaction – with community and government antipathy to issues affecting PWID impacting on funds allocated to resource HCV testing, treatment and care [82,83].

### Housing

There is a clear association between homelessness and HCV antibody positivity in countries such as UK, Canada and USA [84-87]. Homeless PWID tend to have high support needs, yet may face additional barriers when trying to access services [38,86]. Not having an address can, for example, pose problems when registering for income support or with a general practitioner, which can impact on treatment access and self care more generally. While there is scant research on the influence of accommodation circumstances on decisions regarding HCV treatment uptake, homelessness and unstable housing have been identified as barriers to uptake [17,45,61]. Unsurprisingly, PWID can be reticent to take on a treatment with potentially significant side effects while unstably housed [39,45,61].

HCV treatment providers generally consider homelessness or unstable housing to be a signifier for unmanageability and a contraindication for treatment consideration [14,88]. Yet, notable exceptions exist. Services in London [60] and Vancouver [89], for example, have successfully commenced HCV treatment with homeless and unstably housed PWID, by providing outreach services to distribute treatment [60]; offering weekly interferon injections and directly observed therapy at OST prescribing services [89]; and/or providing small fridges for people in hostels and other unstable accommodation in which to store interferon [61]. Treatment adherence among unstably housed PWID has been reported favourably [60]. Taken together, the capacity of unstably housed individuals to cope with the potential rigors of treatment is critical to assessment, including ascertaining the additional supports required.

### Geographic access

For PWID living in rural areas, transport costs and limited choice of health care practitioners, coupled with social isolation and stigma can constitute barriers to HCV screening and management [29,90]. Studies in France have found that poor geographic access to primary healthcare can have a negative influence upon HCV screening uptake [91,92]. A similar study in Scotland found stronger associations between socioeconomic deprivation and limited HCV testing uptake than those related to geographic access. Importantly, however, geographic access was found to affect people on OST more than non-OST participants [78]. Limited geographic mobility can also impact on PWID in large centres, such as London and Sydney, who may be reticent to travel outside of their known area to access HCV testing and treatment [39,60]. Money making, drug procurement, and/or OST dosing demands as well as transport costs and transport access create incentives for PWID not to leave their geographic area to attend non-acute health care appointments [27,39,45]. A Dublin based qualitative study found that convenience was one of the most important facilitators to treatment appointment attendance, with geographic distance from the hospital discouraging attendance [40].

### Criminalisation

The detrimental impact of the criminalisation of PWID on HIV treatment access and adherence is well documented [24,25], but more speculative in relation to HCV treatment access. Evidence more broadly suggests that drug policies emphasising criminalisation can adversely affect treatment access through the confiscation of medications by police, reluctance among PWID to seek help, and the interruption of treatment following arrest, detention or incarceration [23,24]. Often the effects of drug policies emphasising criminalisation may be indirect in relation to HIV or HCV treatment access. For instance, engagement with drug treatment, and especially OST, can enhance HCV treatment access, uptake and adherence among PWID [93], as it does with HIV treatment [94,95], but is limited in many countries, especially in the east of Europe, and most obviously in Russia where it is legally prohibited.

A consequence of the criminalisation of PWID is the risk of their incarceration. HCV is endemic in prison populations globally [96], with recorded prevalence among inmates as high as 57/58% (Australia, Greece) [98]. HCV treatment availability in prisons is uneven [90,96], with barriers to treatment access including: limited access to diagnostic tests, biopsy requirements and delays; abstinence eligibility requirements; prohibitive costs to delivery; and lack of infrastructure and funding [90]. In countries where HCV treatment is available in prisons, such as the UK, USA, Canada, France and Australia, there can be additional difficulties with: taking medications into prison; obtaining consistent clinical monitoring, medical support and follow-up; biopsy requirements; treatment interruptions due to prison transfers, intake and release; lack of aftercare; and limited understanding of HCV treatment by prison staff [40,77,96-99]. There are, however, documented examples of successful HCV treatment provision in prisons, resulting in rates of viral response comparable to treatment in the community [96,98,100].

### Health care systems

The highly compartmentalised nature of health care systems can create a barrier to comprehensive care for PWID whose needs are complex and may span multiple domains, such as drug dependency treatment, acute health care (wounds and infections), psychiatry and hepatology. Hospital-based HCV treatment is often not ideally suited to PWID due to: geographic distance; referral-associated delays [40]; inflexible appointment policies; lengthy waiting times [40,45,61,99,101]; limited infrastructure and psychosocial supports [46,90]; abstinence requirements [40,45]; and prejudicial attitudes of some staff to PWID [40,102]. Barriers to HCV treatment access for PWID include a lack of continued engagement in medical care at the same site [37,61], with some PWID experiencing a lack of consistency in the management and monitoring of their HCV [8], and an accompanying confusion about HCV symptoms, test results and status [8,9]. Navigating health care systems and unfamiliar hospital settings can be daunting for PWID, particularly for those who may have had previous negative experiences with providers [8,45,74].

Health systems' information sharing and confidentiality structures (private versus open spaces, clinics with separate entrances, information sharing protocols) may also perpetuate perceptions of stigma and negative treatment experiences [8,40,103]. Organisational information sharing restrictions can also pose a barrier to adequate care. For example, a qualitative Australian study found that many health care workers, primarily nurses, chose not to disclose knowledge of their HCV treatment patients’ drug using practices to specialist physicians. This was often in order to protect the patient, but could place additional responsibility on nurses and social workers for a patient’s wellbeing, whilst keeping specialist physicians in the dark about treatment successes in the context of active drug use [104].

Communication difficulties between patients and specialists are a common finding in the HCV treatment literature. Research participants have reported feeling unprepared for HCV treatment and experiencing more severe and varied side-effects than they had been led to expect by medical providers [33,34,46]. Reasons for poor communication include: physician’s inadequate explanation of treatment side-effects; use of medical jargon; insufficient duration of consultation; and lack of time and minimal attention to patient concerns [31,33,34,40,44,46,74]. Training in providing care for, and addressing issues relevant to, PWID is often lacking or only superficially provided to HCV treatment providers which may exacerbate unrealistic expectations and communication difficulties [72]. Pre- and post-HCV test counselling has been identified as an area requiring skills development [42,105], and there is a need for physicians to be responsive to patients’ reports of adverse treatment effects [72]. An obstacle to communication that has received little attention to date is language barriers between some immigrants and minority ethnic groups and their HCV treatment providers [61,106].

### Gender

There is emerging evidence describing how gender mediates the experience of HCV [77,107]. Women may experience stigma associated with injecting drug use and HCV more keenly than men [28,40,77]. Whilst there is a dearth of research investigating how gender affects HCV treatment access specifically [77], studies have found women’s concerns about confidentiality, stigma, treatment side effects and intolerance to impact on their treatment uptake decisions more than men [40,108]. HCV treatment knowledge has also been found to be particularly low among women [12,27], with women having higher HCV treatment refusal and/or premature interruption rates than men [108,109]. One study, however, reports a significantly higher rate of dropout from pre-HCV treatment management among men [110]. Evidence more broadly suggests that women’s treatment access decisions are situated alongside their caring responsibilities [27,61], lack of engagement with services (including due to fear of child removal [61,111,112]), incidences of physical, sexual, emotional violence [111-113], and the demands of funding a regular drug supply, including through sex work [111,112]. There is a need to further investigate how gender shapes barriers and facilitators to HCV treatment access, uptake and adherence.

### Culture

There is scant research exploring how culture, ethnicity and migrant status might impact on treatment access among PWID. Hall and colleagues [114] report that non-white people living with HIV in San Francisco study were less likely to undergo HCV testing and referral than their white counterparts, even though all were in touch with primary health care providers. They offer no hypothesis for this finding, calling for further research to address ethnic disparities in HCV care. Giordano et al. [106] found lower treatment initiation rates in black ethnic minority individuals attending a Canadian HCV clinic than their white counterparts, positing that this may result from physician’s reticence to treat, based on their knowledge of reduced SVR rates in black ethnic populations. Almosio et al. [115] recommend that a ‘social assessment’ should be undertaken prior to commencing treatment which includes noting undocumented migrant status, sedentary or nomadic living conditions, the possibility of relocation or return to the homeland, and the impact of cultural understandings of illness and death on treatment acceptability. They recommend immediate treatment if the individual is likely to return to a country of origin where antiviral drugs are not available. London-based research has found that immigrant PWID are often very motivated to access HCV treatment, yet they and their providers face challenges related to: language barriers; deportation possibilities; lack of benefit access; long working hours and potential coercion – especially for women – to enter into treatment by partners and/or relatives. Recommendations include flexible service provision to allow for inflexible work commitments and, even when relatives and partners are available to translate, the provision of at least one session with a skilled interpreter to ascertain treatment readiness [61].

## Discussion

In keeping with a risk and enabling environment framework, we have sought to map the key domains of social factors affecting HCV treatment access and uptake for PWID. Given the paucity of pooled evidence specific to HCV treatment among PWID, we emphasise the preliminary nature of this exercise. We conclude by addressing the implications of the review for creating an enabling environment for HCV treatment access.

### HCV treatment need is socially situated

HCV treatment need is a relative concern. HCV infection and its treatment are situated inside a context of competing everyday concerns experienced by PWID, many of which appear more pressing [27,28]. Research evidences how poverty, homelessness, the demands of funding and maintaining an illicit drug dependency, fear of arrest and incarceration, needle and syringe access, OST provision and restrictions, managing childcare and possible child removal, stigma and social isolation, distrust of police and health care services, and the resulting self management of acute and ongoing health concerns (such as soft tissue infections, drug withdrawal, overdose and depression) and interpersonal violence can all take precedence over HCV prevention or treatment [27,38-40,61,113].

In recognising HCV treatment need as relative, we caution against unrealistic expectations of treatment uptake. Initiatives to enhance treatment access and uptake among PWID are increasingly promoted in population terms, with modelling studies illustrating the cost effective prevention utility of HCV treatment for PWID [5]. Data demonstrating potential decreases in morbidity, mortality and health system spending are compelling in policy environments where treatment support and care for PWID are generally de-prioritised. Against this impetus, it is important to recognise HCV treatment need as a product of ‘situated rationality’ wherein PWID are positioned as entitled to access any treatment available as well as entitled to defer or refuse such treatment. A population-based impetus to increase treatment access and uptake among PWID may place an unwelcome onus on already marginalised individuals to undertake treatment for which they may not be ready or willing. The promotion of universal treatment uptake (including for prevention effect) in the absence of developing concomitant social and structural interventions is a fragile and at best medium-term strategy. This also runs the risk of locating responsibility for low treatment uptake with affected individuals rather than with the social institutions and conditions generative of treatment access obstacles.

### HCV treatment access requires social intervention

In recognising HCV treatment access decisions as a relative concern, it becomes clear that individual-level concerns are shaped by, as well as reproduced through, a variety of social factors which interact as barriers to accessing treatment. This means that PWID who are both in need and eligible for treatment may be unable to realise their treatment opportunity. We have identified social stigma, housing, criminalisation, health care systems, and gender as key domains in the conditionality of HCV treatment access, and thus also, as important targets for social and structural change. We lack the evidence to document here how, for instance, interventions targeting stigma reduction, stable housing, or systemic changes to treatment delivery, may impact upon HCV treatment access and uptake, and although having noted above some such examples [59-61], identify these as critical to future research. Envisaging HCV treatment for PWID as socially situated implies that access is going to be best enhanced when treatment is designed in a *combination intervention approach* and when delivered through *integrated multidisciplinary models*.

Current operating definitions of ‘combination intervention’ in harm reduction for PWID, such as those promoted by the World Health Organization [116], tend to be narrowly defined primarily around biomedical and behavioural interventions. One specific and well evidenced dimension of HCV treatment combination intervention is the critical role of OST in enhancing HCV treatment access, tolerability and adherence [15,50,117]. It has been recommended, for example, that small increases in methadone doses (10 – 15 mg) can help manage HCV treatment side-effects [118], as well as mitigate against potential relapses to – or exacerbation of – injecting drug use [119]. Low threshold access to OST also enhances the impact of HCV prevention [120,121]. The impact of HIV treatment and prevention interventions are similarly enhanced when delivered in combination with high coverage OST [94,95]. The provision of OST take-home doses has been found to enhance PWID trust and engagement with services, as well as proving beneficial for those experiencing HCV treatment related side effects [45,61]. Recommendations for increased access to OST take-home doses [45,61,122] are however, controversal within policy environments favouring ‘recovery’ from illicit drugs of dependance, which – in countries such as the UK – include service incentivisation to restrict ongoing OST provision [122]. Enhancing access to OST is a structural intervention potentially facilitating HCV treatment access, uptake and outcome for PWID.

We also find that HCV treatment access is also facilitated through a combination of low threshold treatment access alongside the delivery of supports in relation to adherence, treatment literacy, and social welfare. Examples of targeted access support include: HCV treatment provision in OST services [45,60,61,93,123] and in conjunction with GP shared care [14]; relaxed eligibility requirements [14,45,60,61]; and flexible opening hours and appointment times [45,61,124]. Targeted adherence supports include: electronic reminder systems [71]; co-ordination with pharmacies for medication dispensing [124]; directly observed therapy [59]; respectful client-centred continuity of care [40,61,71]; nurse provided interferon injections [40]; improved phlebotomy services [45,61,124], including provision to use external jugular venepuncture [125]; and flexible OST provision, including access to take home doses [126]. Targeted treatment literacy supports include: education for PWID [127], as well as training and support for drug and alcohol staff [128] and primary care providers, including the use of video conferencing [129]. While less frequently documented or evaluated, evidenced targeted social supports include: peer support groups [128,130]; peer-workers integrated into HCV treatment provision [131]; improved psychological services [124]; and assistance with practical problems, such as transportation, accommodation and welfare benefit access [61,72,128]. The combination of social and structural supports facilitating HCV treatment access and engagement cautions against an overly narrow biomedical interpretation of combination intervention.

In parallel with the need to design HCV treatment as part of a combination intervention approach, it appears that optimal conditions for treatment delivery comprise integrating care through multidisciplinary teams [14,57,61,123,124]. The delivery of HCV treatment in drug and alcohol settings is an effective way of facilitating low threshold access to HCV treatment as well as integrating treatment alongside other forms of health and social care [15,50,60,61,117]. Yet recent research also cautions against simply ‘adding on’ HCV treatment to drug and alcohol services that are ill equipped to offer flexible and multi-disciplinary care [132,133]. This research notes the potentially detrimental impact of HCV provision in highly regulated OST clinics and raises concerns about the discriminatory attitude of some drug treatment staff to people with HCV [133,134]. Moreover, ‘one-stop-shop’ models of integrated treatment can run the risk of breaching patient confidentiality concerns regarding their HCV status [39], while pharmacotherapy services which preclude disclosure of current drug use makes using such services as a point of low threshold access for HCV treatment difficult.

## Conclusion

With the efficacy of HCV treatment for PWID well evidenced [13,16,89], yet treatment uptake variable and suboptimal [17,21,22], it is timely to move beyond the evidencing of treatment impact among PWID to also consider targeting the factors which inhibit or facilitate treatment access and uptake. We have made a preliminary attempt to map descriptively the social factors mediating HCV treatment access, but what is needed is further research to systematically generate and pool such evidence to determine how multiple social factors interplay in particular settings. A priority is to move beyond typological description towards building models of treatment access with the capacity to include environmental factors and the scope to inform social intervention responses [135]. The study of the conditionality of HIV treatment access among PWID has some useful parallels [24-26]. We suggest that interventions oriented to creating opportunities for stable housing, stigma reduction and systemic changes in policy and health care delivery have the capacity to play a critical role in enhancing HCV treatment access and uptake for PWID. This suggests a combination intervention approach which does not overly rely on biomedical interventions but which includes social, welfare and structural interventions and which seeks to integrate such care services as much as possible at the point of delivery.

## Competing interests

The authors declare they have no competing interests.

## Authors’ contributions

MH carried out the literature searches, review and manuscript drafting. TR contributed to the review design and provided critical revision of manuscript drafts. Both authors read and approved the final manuscript.
